# Anti-polyelectrolyte and polyelectrolyte effects on conformations of polyzwitterionic chains in dilute aqueous solutions

**DOI:** 10.1093/pnasnexus/pgad204

**Published:** 2023-06-19

**Authors:** Zening Liu, Jong K Keum, Tianyu Li, Jihua Chen, Kunlun Hong, Yangyang Wang, Bobby G Sumpter, Rigoberto Advincula, Rajeev Kumar

**Affiliations:** Center for Nanophase Materials Sciences, Oak Ridge National Laboratory, Oak Ridge, TN 37831, USA; Center for Nanophase Materials Sciences, Oak Ridge National Laboratory, Oak Ridge, TN 37831, USA; Neutron Scattering Division, Oak Ridge National Laboratory, Oak Ridge, TN 37831, USA; Center for Nanophase Materials Sciences, Oak Ridge National Laboratory, Oak Ridge, TN 37831, USA; Center for Nanophase Materials Sciences, Oak Ridge National Laboratory, Oak Ridge, TN 37831, USA; Center for Nanophase Materials Sciences, Oak Ridge National Laboratory, Oak Ridge, TN 37831, USA; Center for Nanophase Materials Sciences, Oak Ridge National Laboratory, Oak Ridge, TN 37831, USA; Center for Nanophase Materials Sciences, Oak Ridge National Laboratory, Oak Ridge, TN 37831, USA; Center for Nanophase Materials Sciences, Oak Ridge National Laboratory, Oak Ridge, TN 37831, USA; Center for Nanophase Materials Sciences, Oak Ridge National Laboratory, Oak Ridge, TN 37831, USA

## Abstract

Polyzwitterions (PZs) are considered as model synthetic analogs of intrinsically disordered proteins. Based on this analogy, PZs in dilute aqueous solutions are expected to attain either globular (i.e. molten, compact) or random coil conformations. Addition of salt is expected to open these conformations. To the best of our knowledge, these hypotheses about conformations of PZs have never been verified. In this study, we test these hypotheses by studying effects of added salt [potassium bromide (KBr)] on gyration and hydrodynamic radii of poly(sulfobetaine methacrylate) in dilute aqueous solutions using dynamic light scattering and small-angle X-ray scattering, respectively. Effects of zwitteration are revealed by direct comparisons of the PZs with the polymers of the same backbone but containing (1) no explicit charges on side groups such as poly(2-dimethylaminoethyl methacrylate)s and (2) explicit cationic side groups with tertiary amino bromide pendants. Zeta-potential measurements, transmission electron microscopy, and ab initio molecular dynamics simulations reveal that the PZs acquire net positive charge in near salt-free conditions due to protonation but retain coiled conformations. Added KBr leads to nonmonotonic changes exhibiting an increase followed by a decrease in radius of gyration (and hydrodynamic radius), which are called antipolyelectrolyte and polyelectrolyte effects, respectively. Charge regulation and screening of charge–charge interactions are discussed in relation to the antipolyelectrolyte and polyelectrolyte effects, respectively, which highlight the importance of salt in affecting net charge and conformations of PZs.

**Significance Statement**

Understanding and controlling conformations of polyzwitterions (PZs) in aqueous solutions by adding salt are of paramount interest in areas such as antimicrobial materials, antifouling coatings, drug-delivery, membranes, and polymer electrolytes. We show that the polysulfobetaines, a special class of PZs, remain hydrated in salt-free conditions, contrary to an expectation of globular conformation due to attractive dipole–dipole interactions. Despite having a net positive charge in the solutions, the polysulfobetaines do not exhibit typical polyelectrolyte behavior like two diffusive modes in dynamic light scattering and a peak in small-angle X-ray scattering. Added salt affect the net charge and electrostatic interactions to change chain conformations in a nonmonotonic manner so that both antipolyelectrolyte and polyelectrolyte effects are observed with an increase in the salt concentration in the solutions of the same PZs. Overall, we show that controlling the salt concentration is an effective strategy to tune net charge and conformations of the PZs.

## Introduction

Polyzwitterions, a special class of polyampholytes (1–3), have been studied extensively due to their relevance for various applications such as antifouling coatings (4–6), low-friction materials for synovial joints (7), drug-delivery systems based on coacervation (8), and solid-state batteries (9). Zwitterionic monomers carrying both positive and negative charges in polyzwitterions impart many properties to the materials based on them. These properties include enhanced dielectric constant resulting from an increased dipole moment of monomers (10), enhanced ionic conductivity when doped with salt (9), salt-specific solvation in aqueous solutions due to counterion adsorption, and pH responses (4–6). Due to their poor solubility in water (11–14), salt is used for many applications to improve processing and solubility. A number of studies have focused on understanding the effects of added salt on miscibility of polyzwitterions. It is generally accepted that salt ions can either screen dipole–dipole interactions or adsorb on zwitterionic monomers, both of these effects lead to an improved solubility in water (3, 2, 14). It is expected that a long polyzwitterion chain in dilute aqueous solution attains a globular conformation and will swell with the addition of salt. The swelling of a polyzwitterionic chain with the addition of salt has been observed in experiments (15–17) and is opposite of the behavior exhibited by a polyelectrolyte chain. Such an effect of salt on a polyzwitterionic chain is called the “antipolyelectrolyte effect.”

In general, an improved solubility of polymers in a solvent with the addition of salt implies that chain conformations must be changing due to changes in solvent quality. For example, an improved solubility of polyzwitterions due to addition of salt in water implies that the chains may be transitioning from a globule-like state in salt-free aqueous solutions (i.e. a poor solvent-like condition) to a swollen coil-like state in salty solutions (i.e. a good solvent-like condition). However, the effects of added salt on conformations of a polyzwitterionic chain cannot be described in terms of the solvent quality due to the fact that water and salt ions can affect net charge on a polyzwitterionic chain via protonation of zwitterionic monomers and adsorption of the ions on to the monomers, respectively. For controlling many properties of polyzwitterions in solutions, it is imperative that we understand connections between charge regulation and chain conformations in salt-free and salty conditions. Currently, nature of their conformations (i.e. globular, coil-like, or rod-like ) and effects of added salt on these conformations remain largely unknown. Analogies have been drawn by considering polyzwitterions as synthetic analogs of intrinsically disordered proteins (IDPs) and various conjectures have been made about conformations of these chains in dilute solutions. This leads to ambiguities in not only determining overlap concentrations of polyzwitterions but also understanding complexation of these chains with polyelectrolytes, which is relevant in many biomedical applications (18).

In addition to chain conformations and their modulation by added salt, there is also great interest in understanding dynamics of polyampholytes (19–21). Classical works on dynamics of a single neutral chain (i.e. without explicit charges) (22–24) have revealed the importance of chain connectivity, hydrodynamic interactions, and molecular weight dependence on various macromolecular properties such as viscosity and diffusion. The introduction of explicit charges along the chains and presence of salt ions lead to additional effects such as charge regulation, which are not captured by the classical models for understanding dynamics of polymer chains. In particular, charge regulation of polyzwitterions in aqueous solutions with added salt has a major effect on dynamics of polyzwitterion segments, which affect their rheological and complexation with polyelectrolytes. Other phenomena such as the kinetics of chain collapse (25), electrophoresis in solution (26), translocation dynamics through pores (27, 28), as well as motion under externally applied fields (29–32) are also expected to be affected by the charge regulation. Overall, the dynamics of polyzwitterionic segments and chains remain poorly understood due to complications arising from explicit charges, charge regulation, and electrostatic correlation effects (33, 34).

In this work, we study the effects of added monovalent salt, potassium bromide (KBr), on the radius of gyration and hydrodynamic radii of a polyzwitterion [a poly(sulfobetaine)] in dilute aqueous solutions. This salt was chosen in an effort to reduce types of “free” ions and to complement previous (4) and our ongoing studies related to complexation of the polyzwitterions with cationic polyelectrolytes carrying bromine counterions. For understanding the effects of zwitterionic monomers, we have also studied salt-free solutions of the cationic polyelectrolyte and a polymer without any explicit charges on monomers. For comparison purposes, the polyzwitterion and the polyelectrolyte were synthesized by modifications of pendant groups in poly(2-dimethylaminoethyl methacrylate) (termed as “parent polymer”) so that the polyzwitterions, the polyelectrolytes and the parent polymers have the same length of the main-chain backbone. For characterizing charge regulation of the polyzwitterions, resulting from protonation of charged groups in salt-free and binding of counterions in the presence of the salt, we have used zeta-potential measurements along with density functional theory (DFT)-based simulations. Chain conformations in solutions are probed using small-angle X-ray scattering (SAXS) experiments. Complimentary dynamic light scattering (DLS) measurements are done to probe hydrodynamic radii of the polyzwitterions. SAXS and DLS results are used to develop a picture about the effects of salt on conformations of polyzwitterions in dilute aqueous solutions. It will be shown that binding of cations (protons and potassium) to zwitterionic monomers leads to a net positive charge on the polyzwitterions. An increase in the net positive charge with added salt and the screening of electrostatic interactions are suggested to cause the salt-dependent conformational changes.

## Results

**Fig. 1.**
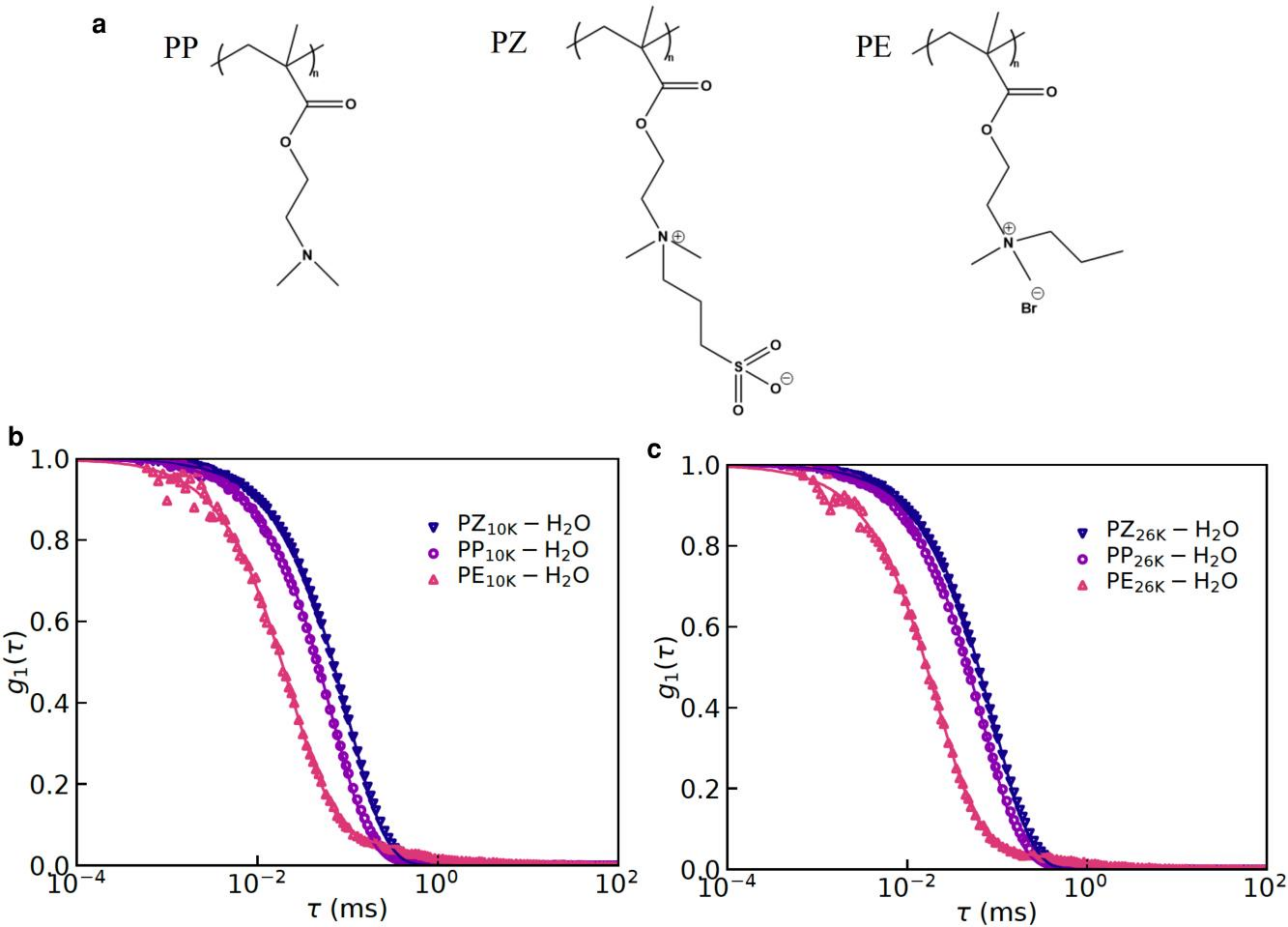
a) Chemical structures of the three types of polymers studied in this work, where PP, PZ, and PE are abbreviations for parent polymer, polyzwitterion, and polyelectrolyte, respectively. DLS results for salt-free solutions containing b) 10 K and c) 26 K PDMAEMA (i.e. PP${}_{10\;K}$-H${}_{2}$O/PP${}_{26\;K}$-H${}_{2}$O ≡ PDMAEMA10k/PDMAEMA26k), polyzwitterions (i.e. PZ${}_{10\;K}$-H${}_{2}$O/PZ${}_{26\;K}$-H${}_{2}$O ≡ PDMAEMA10k-zwitterion/PDMAEMA26k-zwitterion), and polyelectrolytes (i.e. PE${}_{10\;K}$-H${}_{2}$O/PE${}_{26\;K}$-H${}_{2}$O ≡ PDMAEMA10k-electrolyte/PDMAEMA26k-electrolyte) at the scattering angle of $34^{\circ }$.

We have synthesized polyzwitterions and polyelectrolytes (polycations) by modifying the pendant groups of the parent polymers, poly(2-dimethylaminoethyl methacrylate) (PDMAEMA), with either 1,3-propanesultone or 1-bromopropane, respectively. These modifications are highly efficient, evidenced by ${}^{1}$H NMR spectra (see Fig. 1 for the chemical structures, and Figs. S1 and S2 in the Supplementary Material for the spectra). Based on the 100% conversion of the pendant groups, molecular weights of modified materials were calculated and summarized in Table 1. These polymers are named on the basis of the molecular weight of parent polymers and the modification. For example, PDMAEMA10k-electrolyte and PDMAEMA10k-zwitterion are the polyelectrolytes and the polyzwitterions obtained after modifying ∼10 kg/mol PDMAEMA, respectively. While discussing results, a shorthand notation is used so that PP10K, PZ10K, and PE10K mean PDMAEMA10k, PDMAEMA10k-zwitterion, and PDMAEMA10k-electrolyte, respectively. Here, PP, PZ, and PE are abbreviations for parent polymer, polyzwitterion, and polyelectrolyte, respectively.

**Table 1.** Molecular weight information of parent polymers, polyzwitterions and polyelectrolytes studied in this work.

Polymer	$M_{n}$ (kg/mol)	$N_{m}$	Ð
PDMAEMA10k	10.3	65	1.15
PDMAEMA26k	26.4	168	1.13
PDMAEMA10k-electrolyte	18.2	65	1.15
PDMAEMA26k-electrolyte	47.0	168	1.15
PDMAEMA10k-zwitterion	17.7	65	1.13
PDMAEMA26k-zwitterion	45.8	168	1.13

$M_{n}$ is number-average molecular weight, $N_{m}$ is degree of polymerization, and Ð is dispersity. Molecular weights of the polyzwitterions and the polyelectrolytes were calculated based on 100% conversion of the pendant groups.

### Effects of zwitteration and salt on hydrodynamic radius

We have measured the intensity–intensity correlation function (ICF) (Eq. 1) of the scattered light (with wavelength, λ=633 nm) at eight scattering angles ($34^{\circ },50^{\circ },68^{\circ },84^{\circ },102^{\circ },118^{\circ },136^{\circ },\;\mathrm{and}\;152^{\circ }$). In Fig. 1, we have presented the ICF for salt-free solutions containing the parent polymers, polyzwitterions, and polyelectrolytes at the same mass concentration of 10 mg/mL and at the scattering angle of $34^{\circ }$. It can be readily observed that the ICFs for the polyzwitterions and the polyelectrolytes decay at the slowest and the fastest rates, respectively, for short lag times (i.e. τ<0.1 ms). The polyelectrolytes exhibit a typical two-step decay (35, 33), which is associated with “free” chains and “aggregates” in solutions. The fact that the polyzwitterions did not exhibit a similar two-step decay implies that these polymers do not behave like typical polyelectrolytes. Nevertheless, dynamics of concentration fluctuations in polyzwitterions is found to be the slowest. Also, this result is the same for the two molecular weights studied in this work and shown in Fig. 1. These results are counter-intuitive because the polyzwitterions can undergo protonation/deprotonation in salt-free aqueous solutions, which can lead to a net positive charge on the chains. In this sense, the polyzwitterions should behave like the polyelectrolytes. In retrospect, these results imply that the net charge on the polyzwitterionic chains in the salt-free solutions is too small to exhibit a typical polyelectrolyte behavior.

**Fig. 2.**
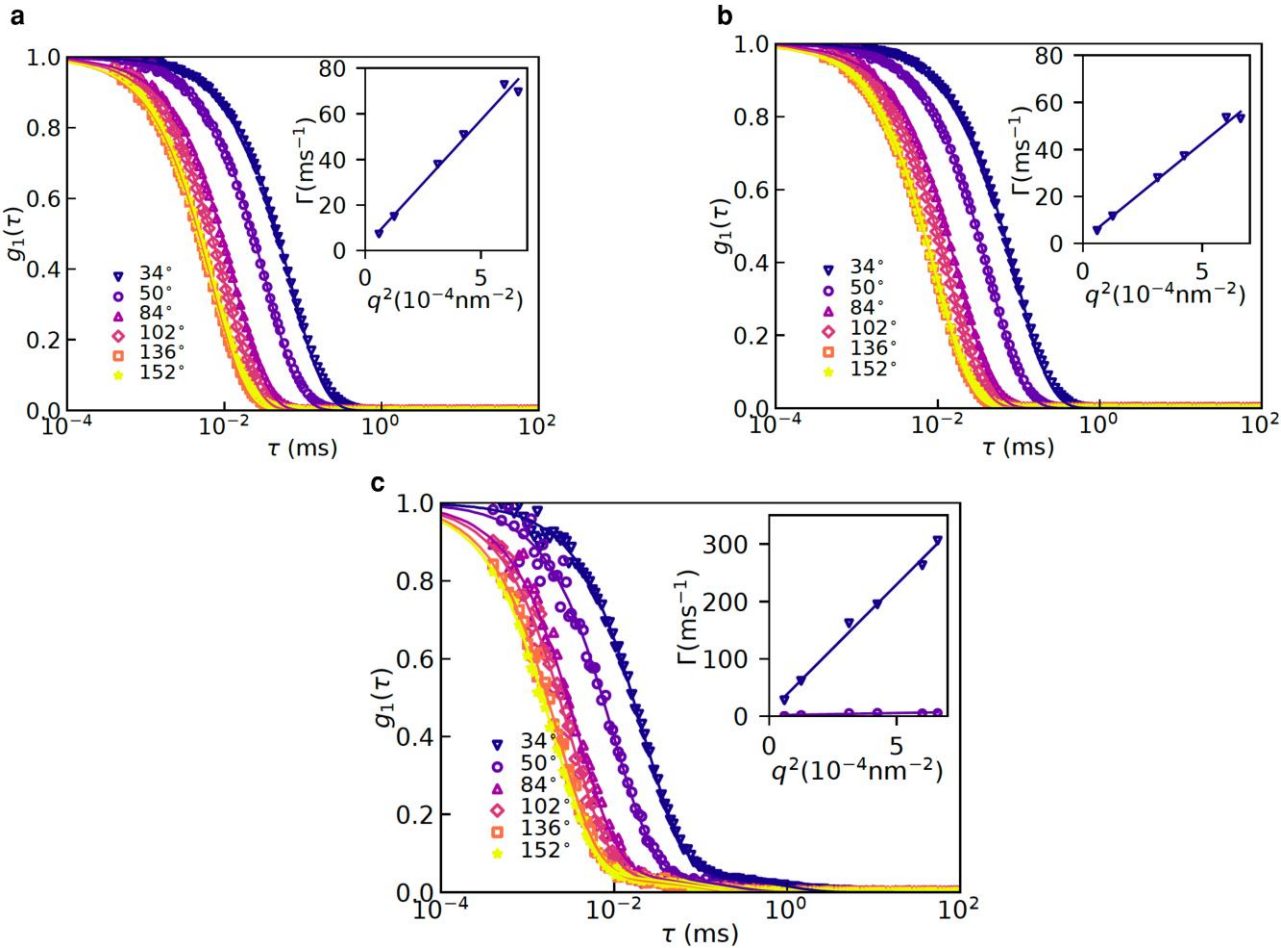
Multiangle DLS results for salt-free solutions containing 26 K parent polymer (PP) PDMAEMA26k, PDMAEMA26k-zwitterion, and PDMAEMA26k-electrolyte are shown in panels a), b), and c), respectively. DLS was measured for eight angles but data for only six angles are shown here to improve clarity of the figures.

**Fig. 3.**
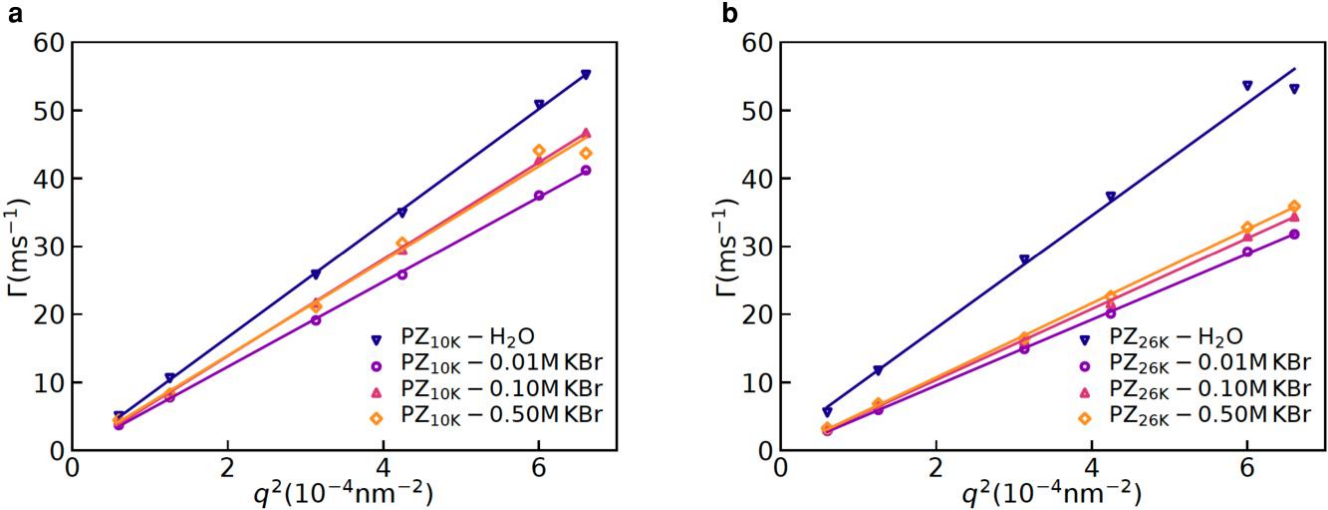
Decay rates at different salt concentrations for solutions containing a) PDMAEMA10k-zwitterion and b) PDMAEMA26k-zwitterion. A nonmonotonic effect of the salt concentration on the decay rates is observed for both of the molecular weights so that the decay rate first increases and then decreases with an increase in the salt concentration.

For the purpose of estimating hydrodynamic radii from the DLS data, we first established diffusive character of the dynamics probed in these experiments. This is done by plotting the decay rates, Γ(q), extracted from the ICFs (see the Materials and Methods section for details) shown in Fig. 2 as a function of $q^{2}$ and observing a linear relation between Γ(q) and $q^{2}$. Indeed, linear relations among Γ(q) and $q^{2}$ were observed (see the insets in Fig. 2 and Figs. S3–S11 in the Supplementary Material), which established diffusive character of the concentration fluctuations probed using the DLS. Now, using the Stokes–Einstein equation (Eq. 2), the hydrodynamic radii ($R_{h}$) can be readily obtained, using η as the viscosity of water at the room temperature. Before we present results for the effects of zwitteration on the hydrodynamic radii, we discuss the effects of added salt on it and the radius of gyration. These additional measurements provide a complete picture about effects of salt on the chain conformations of polyzwitterions in aqueous solutions. In particular, the decay rates for the polyzwiterionic solutions at different concentrations of KBr are shown in Fig. 3 for the two different molecular weights. Fig. 3 shows that an increase in the concentration of KBr led to a moderate decrease followed by an increase in the decay rates for the both, PZ${}_{10\;K}$ and PZ${}_{26\;K}$. Specifically, there was a significant effect of the salt on the decay rates for adding small amount of KBr (0.01 M) and a weak effect of added salt on increasing the concentration up to 0.5 M.

Based on previous reports (14, 13), salt can affect chain conformations in two manners. First, salt ions can adsorb asymmetrically on to the zwitterionic monomers, leading to either an increase or decrease of the net charge of polyzwitterionic chains. Second, salt ions can screen all electrostatic interactions including charge–charge, charge–dipole, and dipole–dipole. These effects of added salt on polyzwitterions need to be contrasted with polyelectrolytes, where theoretical calculations (36, 37) highlight that an increase of salt concentration can lead to enhanced charging of the polyelectrolytes. In order to understand how the added salt KBr affect the confomations of the polyzwitterions, PZ${}_{10\;K}$ and PZ${}_{26\;K}$, we have used the SAXS for the purpose of estimating the radius of gyration and learning its relations with the zwitteration and added salt concentrations.

### Effects of salt on radius of gyration and chain conformation

**Fig. 4.**
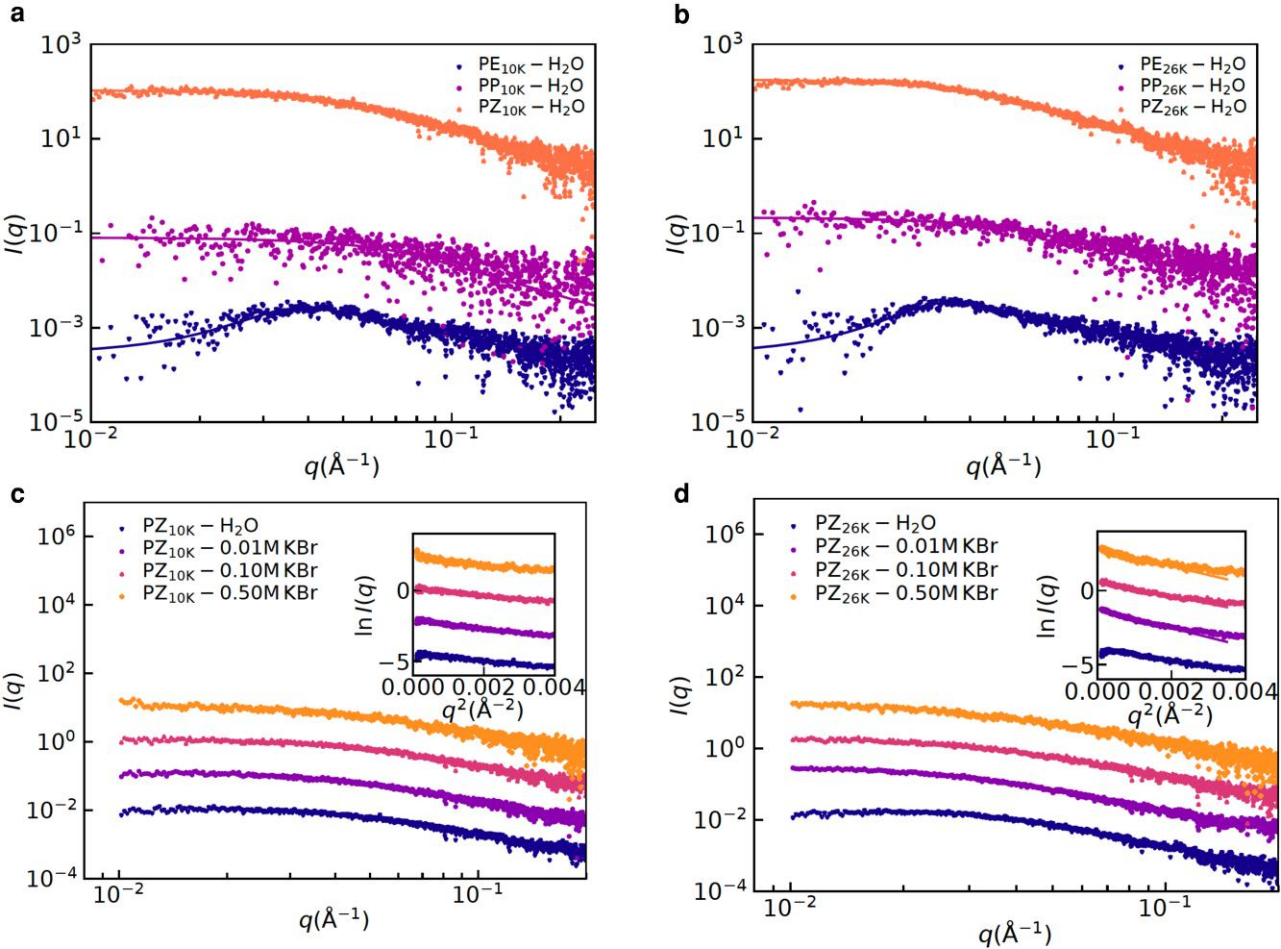
SAXS results for understanding effects of zwitteration for the polymers obtained by modification of PDMAEMA10k and PDMAEMA26k are shown in panels a) and b), respectively. Lines are fits using the models described in the main text. Effects of added KBr on the SAXS for the aqueous solutions containing PDMAEMA10k-zwitterion and PDMAEMA26k-zwitterion are shown in panels c) and d), respectively. Insets in panels c) and d) show the Guinier plot (lnI(q) vs $q^{2}$) and Guinier (38) fits to estimate radii of gyration, see Eq. 3. Curves were shifted vertically for clarity.

Fig. 4 shows the typical results of background subtracted scattering intensity I(q) as a function of wavevector *q* obtained from SAXS experiments on aqueous solutions containing the polymers at various ionic strengths. Unlike PE${}_{10\;K}$ and PE${}_{26\;K}$ clearly exhibiting polyelectrolyte peaks, the polyzwitterions showed typical Guinier-type scattering shoulders of spherical polymer coils with no interchain correlation. The concentration normalized SAXS curves of PZ${}_{10\;K}$ and PZ${}_{26\;K}$ falling onto the respective master curves confirm that the SAS curves are essentially from the isolated PZ${}_{10\;K}$ and PZ${}_{26\;K}$ coils with no intercoil aggregation in the solutions, see Fig. S12a and b in the Supplementary Material. Also, the concentration independence of the SAXS form factor implies that the concentration regime of 2, 5 and 10 mg/mL PZ solutions were in a dilute regime. The correlation peaks of PE${}_{10\;K}$ and PE${}_{26\;K}$ were associated with the correlations between the semirigid polyelectrolytes chains, where the correlation lengths of PE${}_{10\;K}$ and PE${}_{26\;K}$ chains obtained from the SAXS model fit using the polymer reference interaction site model (PRISM) (see the Supplementary material for the details) were found to be 52.5 and 58.8 Å, respectively. Also, the correlation lengths were found to increase with decreasing PE concentrations, see Table S1 and Fig. S12a and S12b in the Supplementary Material. Differently from polyelectrolytes chains, the results of polyzwitterions indicating isolated single polymer coil behavior without intercoil interaction are in qualitative agreement with the DLS measurements showing a single-step decay for the polyzwitterions. Also, addition of salt does not affect this behavior of polyzwitterions, i.e. in panels Fig. 4c and d, we do not observe any polyelectrolyte peak with the additions of the salt to PZ${}_{10\;K}$ and PZ${}_{26\;K}$, respectively.

**Fig. 5.**
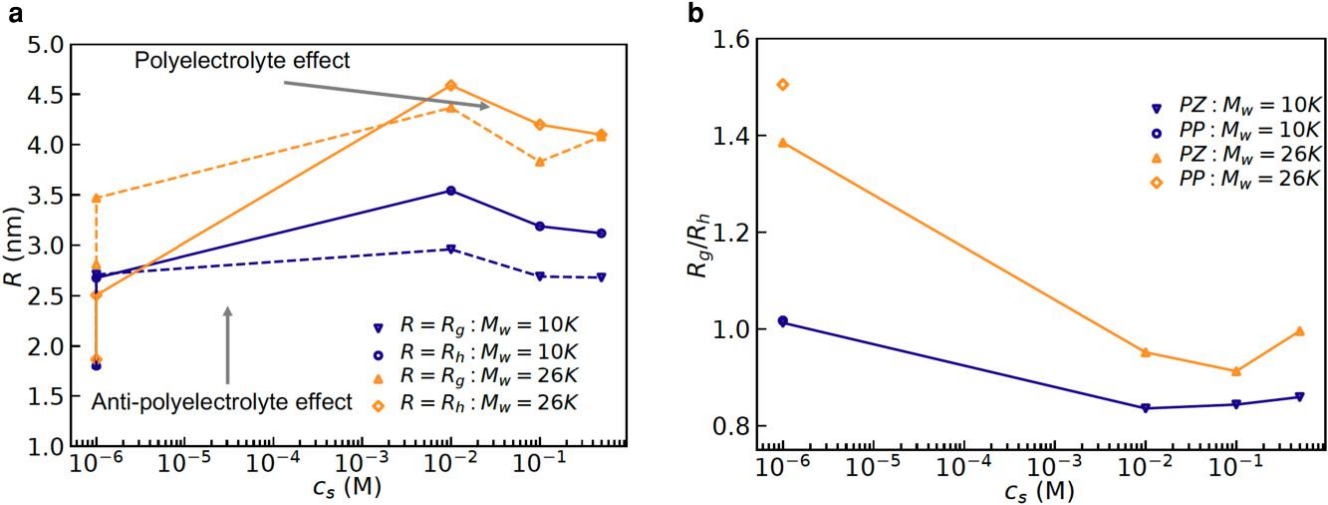
a) Effects of added KBr on $R_{g}$ and $R_{h}$ of PDMAEMA10k-zwitterion and PDMAEMA26k-zwitterion. b) Effects of added salt on the ratio $R_{g}/R_{h}$ is plotted for both the polymers. “Salt-free” solutions are assumed to contain $10^{-6}$ M salt for this semilog plot.

We have estimated the radius of gyration ($R_{g}$) from such SAXS data using the Guinier analysis (i.e. after plotting lnI(q) as a function of $q^{2}$). These results are shown in Fig. 5 along with the hydrodynamic radii estimated using the DLS. We found that both the $R_{g}$ and $R_{h}$ exhibit nonmonotonic behavior with an increase in the salt concentration i.e. they increase first and then decrease to a value higher than its value in the salt-free conditions (cf. Fig. 5a). The increase and the decrease in $R_{g}$ or $R_{h}$ are clear manifestations of the antipolyelectrolyte and the polyelectrolyte effect, respectively. These effects are present in the polyzwitterions independent of the molecular weights. For inferring shape of these chains in the solutions, we have plotted the ratio $R_{g}/R_{h}$ in Fig. 5b. Noting that $R_{g}/R_{h}=1.59$ and 0.77 correspond to a Gaussian coil in good solvent and a globule, respectively, Fig. 5b shows that the polyzwitterions in salt-free solutions behave like the Gaussian coils in good solvent. Also, Kratky plot ($Iq^{2}$ vs *q*) of the SAXS curves of the salt-free PZ solutions exhibiting clear plateau at high-*q* confirmed the Gaussian(or Gaussian-like) conformations. In other words, these chains are not globular in water, and remain hydrated, in contradiction to a collapsed conformation expected on the basis of strong intrachain dipole–dipole interactions. Here, we should point out that an estimated (39) dipole moment of the zwitterionic monomer (based on the electronic Density Functional Theory) is 15.2 Debye, which is almost 10 times that of water (40) (1.8 Debye). Based on these estimates of the dipole moments, it is expected that the monomers should aggregate with each other rather than the solvent. Nonmonotonic effects of the added salt can be rationalized by interplay of two phenomena: (1) selective binding of one of the ions of the salt on to the zwitterionic monomers, leading to expansion of a polyzwitterionic chain and (2) screening of electrostatic interactions (i.e. charge–charge, charge–dipole, and dipole–dipole) by the added salt. However, selective binding of the ions to zwitterionic monomers has to be weak enough so that a polyzwitterionic chain does not behave like a typical polyelectrolyte. Furthermore, both screening of dipole–dipole interactions and selective binding of ions to zwitterionic monomers can lead to increase of the radius of gyration. For the purpose of distinguishing between the two mechanisms (i.e. selective binding and screening) for an increase in $R_{g}$, we have used ab initio molecular dynamics simulations, zeta-potential, and transmission electron microscopy (TEM) measurements.

**Fig. 6.**
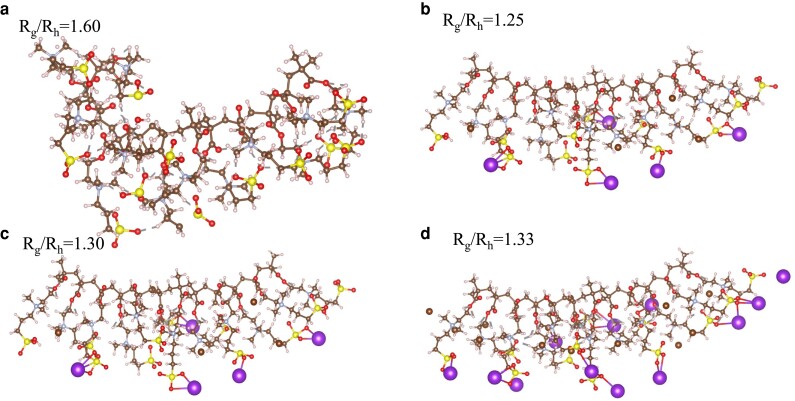
Snapshots of a polyzwitterion chain taken from an ab initio molecular dynamics simulation in solvent (not shown) with varying amounts of KBr salt. These snapshots are taken after each system had reached a steady state as determined by a converged radius of gyration. The potassium ions are shown as the largest sphere. Atom colors are: C brown; hydrogen white; oxygen red; sulfur yellow; potassium purple.

**Fig. 7.**
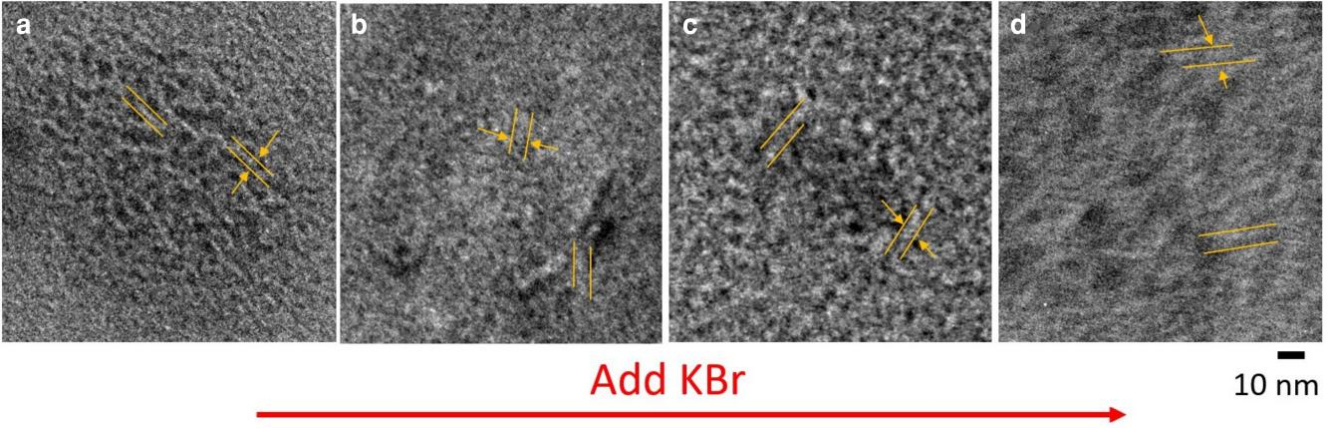
TEM images for PDMAEMA26k-zwitterion drop-casted from 10 mg/mL aqueous solutions containing a) no added salt, b) 0.01 M KBr, c) 0.1 M KBr, and d) 0.5 M KBr.

Snapshots of a polyzwitterionic chain with added KBr and simulated using density functional tight-binding (DFTB) are shown in Fig. 6. These snapshots show that potassium binds to the sulfonated groups on the zwitterionic monomers, leading to conformational changes. Similarly, our zeta-potential measurements (see Table 2) reveal that the polyzwitterionic chains acquire weak positive charge in low salt conditions, which confirm that indeed selective binding of cations (protons and potassium) leads to Gaussian coil-like conformation and an increase in its radius of gyration at low salt concentrations (i.e. <0.01 M). Net zeta potential of the polyzwitterionic chains are smaller than that of the polycations studied here, and that may be the reason behind the lack of a polyelectrolyte peak in SAXS. Screening the electrostatic interactions at higher salt concentrations leads to the decrease in the radius of gyration of the polyzwitterionic chains. These results are in qualitative agreement with the calculations based on a uniform expansion model (14) and TEM measurements. Specifically, Fig. 7 shows the TEM results of the 26k series as a function of salt concentration. The dark and light regions reflect the mass-thickness contrast of the corresponding structures, with denser packing or larger thickness contributing to darker areas. Based on ten measurements, the sample without salt exhibit finer structures with feature sizes down to 1.3±0.5 nm, and as the salt concentration increases, feature sizes increase dramatically to 3.5±1.2 nm. Furthermore, in the samples with salt complexes, elongated structures are obvious. These correlate well with our simulations and scattering results. Overall, this work shows that added salt can lead to either antipolyelectrolyte or polyelectrolyte effects depending on the concentration of the added salt, net charge, and conformations of polyzwitterions in salt-free aqueous solutions. In Ref. (17), it was shown that the antipolyelectrolyte effect is nonuniversal in nature and depends on the specificity of salt. Our work suggests that net charge of the polyzwitterions in salt-free solutions plays a decisive role in affecting the antipolyelectrolyte behavior, which verifies similar conjectures made in the literature (14, 16).

**Table 2.** Zeta potential of parent polymers, polyzwitterions and polyelectrolytes in 10 mg/mL salt-free aqueous solutions.

Polymer	ζ (mV)
PDMAEMA10k	11.15
PDMAEMA26k	3.05
PDMAEMA10k-electrolyte	64.60
PDMAEMA26k-electrolyte	41.03
PDMAEMA10k-zwitterion	18.54
PDMAEMA26k-zwitterion	14.97

## Conclusions

The polyzwitterion chains in salt-free dilute aqueous solutions stay in Gaussian coil form, despite being positively charged. However, the chains do not exhibit a polyelectrolyte peak in SAXS and two modes in DLS. These results indicate that the polyzwitterionic chains are weakly charged, which is confirmed by zeta-potential measurements. Addition of KBr leads to increase (antipolyelectrolyte effect) in gyration and hydrodynamic radii followed by a decrease (polyelectrolyte effect) resulting from selective binding of potassium to the sulphonted groups on the zwitterionic monomers and screening of electrostatic interactions, respectively. The antipolyelectrolyte effect is observed at lower salt concentrations (≤0.01 M). For higher salt concentrations, the polyelectrolyte effect sets in and eventually, the chain conformations become insensitive to the salt concentration. Although the antipolyelectrolyte effect is nonuniversal, we expect the net charge of polyzwitterionic chains in salt-free solutions to play a decisive role in affecting the behavior of polyzwitterions and hence, point out at the use of salt to control net charge and conformations of the polyzwitterion chains. We envision the use of salt-induced changes on chain conformations and net charge in designing processing protocols for mixing and dissolving various polyzwitterionic materials.

## Materials and methods

### Materials

All reagents were used as received from suppliers without further purification unless otherwise noted. 2-Dimethylaminoethyl methacrylate was purified by passing through the basic Al${}_{2}$O${}_{3}$ column to remove the inhibitor.

### Synthesis of poly(2-dimethylaminoethyl methacrylate)s

Poly(2-dimethylaminoethyl methacrylate)s (PDMAEMA) were synthesized by atom transfer radical polymerization using ethyl 2-bromoisobutyrate as the initiator and 1,1,4,7,10,10-hexamethyltriethylenetetramine (HMTETA) as the ligand. In a typical procedure, purified 2-dimethylaminoethyl methacrylate (20.10 g, 127.85 mmol, 156 eq), HMTETA (0.21 g, 0.91 mmol, 1 eq), ethyl 2-bromoisobutyrate (0.16 g, 0.82 mmol, 1 eq), copper (II) chloride (0.01 g, 0.07 mmol, 0.1 eq), and 25 mL of toluene were added into a 50 mL flask under the inert atmosphere. After degassing by the freeze-pump-thaw method 3 times, copper (I) chloride (0.07 g, 0.71 mmol, 1 eq) was added to the reaction and degassed again. The reaction was then stirred at 50${}^{\circ }$C for 3 h, followed by quenching in the liquid nitrogen. Purification was achieved by precipitation in hexanes to afford light green/yellow-colored solids as the pure products (61% yield).

### Pendant group modifications of poly(2-dimethylaminoethyl methacrylate)s

Pendant groups of zwitterions (sulfobetaine) were converted from the tertiary amine groups. In the typical procedure, parent polymer (3.0 g, 19.1 mmol, 1 eq), 1,3-propanesultone (4.7 g, 38.2 mmol, 2 eq), and 25 mL of acetonitrile were added into a 50 mL flask. After purging by bubbling with nitrogen, the reaction was stirred at 40${}^{\circ }$C overnight. The obtained polymer was dialyzed in water with 1k cutoff tubing to yield white solids as the pure products (quantitative yield).

A similar procedure was applied to obtain pendant groups of ammonium bromide. Parent polymer (3.0 g, 19.1 mmol, 1 eq), 1-bromopropane (4.7 g, 38.2 mmol, 2 eq), and 25 mL of acetonitrile were added into a 50 mL flask. Then the reaction was purged by bubbling with nitrogen, followed by stirring at 40${}^{\circ }$C overnight. The excess of 1-bromopropane and solvent were removed by high vacuum to offer the pure product as yellow solids (quantitative yield).

### Zeta-potential and DLS measurements

Polymer aqueous solutions were prepared by dissolving polymers into water with or without KBr at room temperature and the polymer solutions were passed through a 0.2 μm Nylon membrane. Zeta potential and complementary DLS measurements were conducted using Zetasizer Ultra from Malvern Instruments Ltd. The samples were measured in a folded capillary cell (DTS1070) at $173^{\circ }$ scattering angle (backscattering) with a He–Ne laser (λ=632.8 nm). The sample cells were thoroughly washed with filtered DI water (filtered through a 0.2 μm cellulose acetate membrane) several times and dried using filtered air. Each sample at polymer concentration of 10 mg/mL was measured over 5 times and the data was obtained by averaging these measurements. All the experiments were conducted at $25^{\circ }$C. We measured the zeta potential in salt-free conditions and 0.5 M KBr. However, measurements were not reproducible in 0.5 M KBr, most probably due to high conductivity of the solutions and nonspherical character of the polymers in the high salt conditions. So, we reported the zeta-potential in salt-free aqueous solutions. Also, we measured the zeta potential using 5 mg/mL aqueous solutions, and quantitatively similar results were obtained.

Multiangle DLS experiments were performed on an ALV compact goniometer system with 7002 Multiple Tau Digital Correlator and Helium–Neon laser (λ=632.8 nm). Prior to the scattering experiment, each sample was filtered through a 0.1 μm poly(vinylidene difluoride) (PVDF) filter. The intensity correlation function (ICF) was collected at eight scattering angles: $34^{\circ }$, $50^{\circ }$, $68^{\circ }$, $84^{\circ }$, $102^{\circ }$, $118^{\circ }$, $136^{\circ }$, and $152^{\circ }$. All the experiments were conducted at room temperature ($T\approx 20^{\circ }$C).

The ICF was modeled by either a single exponential decay or the superposition of two exponential decays:


(1) $$\begin{array}{rl} & \frac{\left\langle I(t)I(0)\right\rangle }{\langle I\rangle^{2}}-1=\gamma [g_{1}(t){]}^{2}\;=\gamma [\phi_{1}\exp\;(-t/\tau_{1})+(1-\phi_{1})\exp\;(-t/\tau_{2}){]}^{2}, \end{array}$$

where $g_{1}(t)$ is the electric field correlation function, γ is the spatial coherence factor, $\phi_{1}$ is the relaxation strength, and $\tau_{i}$ is the characteristic relaxation time. A single exponential function was used for PP and PZ, whereas two exponential functions were necessary for modeling the ICF of PE (35, 33). For a single-chain diffusion process, the measured relaxation τ can be related to the hydrodynamic radius $R_{h}$ through the Stokes–Einstein relation:


(2) $$R_{h}=\frac{k_{B}T\tau q^{2}}{6\pi \eta },$$

where $k_{B}$ is the Boltzmann constant, *T* is the temperature, *q* is the scattering wave number, and η is the solvent (water) viscosity. The scattering wave number is defined by the medium refractive index *n*, incident light wavelength λ, and scattering angle θ as: q=(4πn/λ)sin(θ/2).

### ${}^{1}$ H nuclear magnetic resonance

${}^{1}$ H nuclear magnetic resonance (NMR) spectra were obtained on a Bruker-500 MHz NMR spectrometer at $25^{\circ }$C in deuterated chloroform (CDCl${}_{3}$) (7.27 ppm ${}^{1}$H reference) or deuterated oxide (D${}_{2}$O) (4.79 ppm ${}^{1}$H reference) unless otherwise noted.

### Transmission electron microscopy

Sample solutions were drop cast on a continuous carbon-coated copper grids with 400 mesh (Ted Pella). The grids were then dried with filter paper and sealed away from moisture before experiments. JEOL NEOARM was operated at 80 kV in low beam conditions. A spot size of 5 and low screen brightness was combined to perform TEM studies with minimum radiation damage from the electron beam. Image J was used to extract plot profiles from the TEM images and make measurements on domain sizes. Ten measurements were performed for each sample.

### Small-angle X-ray scattering

Small-angle X-ray scattering (SAXS) measurements were carried out on a Xeuss 3.0 SAXS instrument (Xenocs, France) equipped with a D2+ MetalJet X-ray source (GaKα) (Excillum, Sweden) and Dectris Eiger 2R 4M hybrid photon counting detector, which has an active detecting area of 155.1mm×162.2mm and pixel dimension of 75μm×75μm. The sample to detector distance was 1750 mm and the exposure time was 30 min. The collected 2D SAXS images were circularly averaged and reduced to intensity versus wavevector (*q*), where q=(4πsinθ)/λ, after subtraction of background scattering.

The SAXS data was fitted using the Guinier–Porod model as follows (38, 41, 42):


(3) $$I(q)=G\exp\;[-q^{2}R_{g}^{2}/3]\;\text{for}\;q\leq q_{1}\;(\text{Guinier term}),$$

(4) $$\;I(q)=D/q^{m}\;\text{for}\;q\geq q_{1}\;(\text{Porod term}),$$

where I(q) is the scattered intensity, $R_{g}$ is the radius of gyration, *m* is the Porod exponent, *G* and *D* are the Guinier and Porod scale factors, respectively. For the Guinier and Porod terms to be continuous at $q_{1}$, the following relations are used:


(5) $$\;q_{1}=R_{g}^{-1}(3m/2{)}^{1/2},$$

(6) $$D=G\exp\;[-q_{1}^{2}R_{g}^{2}/3]q_{1}^{m}.$$

### First principles’ simulations

DFTB simulations were used to evaluate the proton affinity for poly(sulfobetaine methacrylate), PSBMA, and its structure at room temperature in a COSMO model (43) of water without periodic boundary conditions. The dielectric constant was taken as 80.2, the molecular mass of water 18 g/mol, and the density was 1 g/cc. DFTB and the extended tight-binding methods enable simulations of relatively large systems and long timescales at a reasonable accuracy but are considerably faster than typical ab initio DFT (44). Prior work has also shown reasonably accurate results for zwitterions (45). We used DFTB version 21.1 with the third-order parameterization for organic and biological systems (3OB) (46) alongside dispersion corrections via the DFTD3 approach to compute the proton affinity and the room temperature dynamics of a 12-mer PSBMA chain in water. Geometry optimization was converged to a maximal force component of 0.0001 eV/Å using the conjugate gradients approach. The converged calculations indicate that protonation of the sulfite groups in PSBMA is probable; a proton affinity of 213 kcal/mol computed as the difference between the total energy of a PSBMA with one sulfite protonated and the energy of unprotonated PSBMA plus a proton in a model water solvent. Additionally, we checked to be sure the computed proton affinity did not show a strong chain length dependence. Finally, simulations where a free proton was added to a simulation of PSBMA showed rapid protonation of a nearby sulfite group. Based on these results, sulfite protonation appears likely and would cause an overall positive charge. Indeed a positive charge was measured by the zeta-potential experiments.

DFTB molecular dynamics simulations of PSBMA at room temperature were carried out using a 1 fs time step and a Nose Hoover thermostat, see Fig. 6 for the molecular structure of the PSBMA chain. The results from those simulations indicated that the sulfite groups remain largely coordinated with H atoms of methyl groups on the quaternary amines. There is coiling of the backbone but the zwitterion side chains stay mainly stretched into water, other than the sulfite tails bending back to interact with a H atoms as mentioned above. We also performed similar calculations for poly(2-dimethylaminoethyl methacrylate) (PDMAEMA) for comparison and note that for PDMAEMA, the quatenary amine groups also have a relatively high proton affinity (185 kcal/mol), and similar to the sulfite groups in PSBMA, appear to become protonated. MD results for PSBMA with added KBr salt indicates mediation of the so-called antielectrolyte effect via breaking up dipole–dipole attractions between the zwitterions which leads to a expanded geometry; an effect that is enhanced with increasing salt concentration (see Fig. 6). To better quantify the effects, we computed the radius of gyration and an approximate hydrodynamic radius or each case based on the results of a MD simulation spanning a timescale of 100 ps. For an approximate measure of the hydrodynamic radius, we used a cylinder that encompassed all the ions and the PSBMA. The radius of gyration had a standard deviation of less than 2 Å for all cases shown in Fig. 6.

## Supplementary Material

pgad204_Supplementary_Data

## Data Availability

All of the data presented in this work is available at https://www.dropbox.com/sh/z5ika3g0v5y2dhe/AADC1uXTszoqt37HZ62b_5q_a?dl=0
