# Supplementary material for: Anti-polyelectrolyte and polyelectrolyte effects on conformations of polyzwitterionic chains in dilute aqueous solutions
Source: PNAS Nexus. 2023 Jun 19;2(7):pgad204. doi: 10.1093/pnasnexus/pgad204 (PMC10323900; doi:10.1093/pnasnexus/pgad204)
Supplement: pgad204_Supplementary_Data [file pgad204_supplementary_data.pdf]

Supporting Materials for: Anti-polyelectrolyte and polyelectrolyte effects on conformations of polyzwitterionic chains in dilute aqueous solutions

Zening Liu,[†] Jong K. Keum,[†,‡] Tianyu Li,[†] Jihua Chen,[†] Kunlun Hong,[†] Yangyang Wang,[†] Bobby G. Sumpter,[†] Rigoberto Advincula,[†] and Rajeev Kumar[*,†]

†*Center for Nanophase Materials Sciences, Oak Ridge National Laboratory, Oak Ridge, TN 37831*

‡*Neutron Scattering Division, Oak Ridge National Laboratory, Oak Ridge, TN 37831*

E-mail: kumarr@ornl.gov

1 ^{1}H NMR spectra

The pendant group modification with 1,3-propanesultone or 1-bromopropane is highly efficient, evidenced by ^{1}H NMR spectra where the shifts of the three sets of protons related to tertiary amines after the reactions. For the parent polymer, protons of methyl groups on -N(CH_3)$_2$ have a chemical shift of 2.3 ppm, and protons of methylene groups near oxygen (-CH_2-O-) and nitrogen (-CH_2-N-) were at 4.0 and 2.6 ppm, separately, which were labeled in Figure S1 and Figure S2. After the modification, they were shifted to 3.3 ppm, 4.6 ppm, and 3.9 ppm. In addition, the new signals of protons related to 1,3-propanesultone and 1-bromopropane had the excepted ratios of integrations with the pendant groups, which further

proved the quantitative conversions of the pendant group modification. ^{1}H NMR spectra of PDMAEMA10k-electrolyte, PDMAEMA26k-electrolyte, PDMAEMA10k-zwitterion, and PDMAEMA26k-zwitterion were illustrated in Figures S1 and S2 , separately.

2 Dynamic Light Scattering

Additional data for the intensity-intensity correlation function obtained from the solutions containing the polymers are shown in Figs. S3- S11.

3 Small Angle X-ray Scattering (SAXS)

3.1 Fitting SAXS from polyelectrolyte solutions

Scattering intensity from polyelectrolyte solutions at a wavevector q can be written in a form[1–3]

$$I(q) \quad = \quad k\frac{P(q,L,b)}{1+\beta\exp(-q^2\xi^2)P(q,L,b)} \tag{S1}$$

where L and b are the contour length and Kuhn length, respectively. $\beta = 1/S(0)-1$ and k is the scaling factor resulting from the contrast. $P(q,L,b)$ is the form factor of a polyelectrolyte chain, which has an explicit expression and can be found in Refs.[1–3]

Table S1: Fit parameters obtained after fitting experimentally measured SAXS data with Eq. S1 for the polelectrolytes, PDMAEM10k-electrolyte and PDMAEM26k-electrolyte.

	PE_{10K}			PE_{26K}		
Parameter	2 mg/ml	5 mg/ml	10 mg/ml	2 mg/ml	5 mg/ml	10 mg/ml
Contour Length,Å	581.20	597.00	559.40	1552.10	1502.30	1426.50
Kuhn Length, Å	10.00	10.30	11.50	10.10	10.90	11.90
β	12.70	15.40	17.30	21.20	37.40	48.80
ξ, Å	95.50	61.20	52.50	98.10	78.30	58.80

Additional SAXS data for the 10 K and 26 K polymers at different concentrations of

polymers (c_p) in water are shown in Fig. S12.

References

(1) Pedersen, J. S.; Schurtenberger, P. Scattering functions of semiflexible polymers with and without excluded volume effects. *Macromolecules* **1996**, *29*, 7602–7612.

(2) Chen, W.-R.; Butler, P. D.; Magid, L. J. Incorporating Intermicellar Interactions in the Fitting of SANS Data from Cationic Wormlike Micelles. *Langmuir* **2006**, *22*, 6539–6548, DOI: 10.1021/la0530440.

(3) Marciel, A. B.; Srivastava, S.; Tirrell, M. V. Structure and rheology of polyelectrolyte complex coacervates. *Soft Matter* **2018**, *14*, 2454–2464.

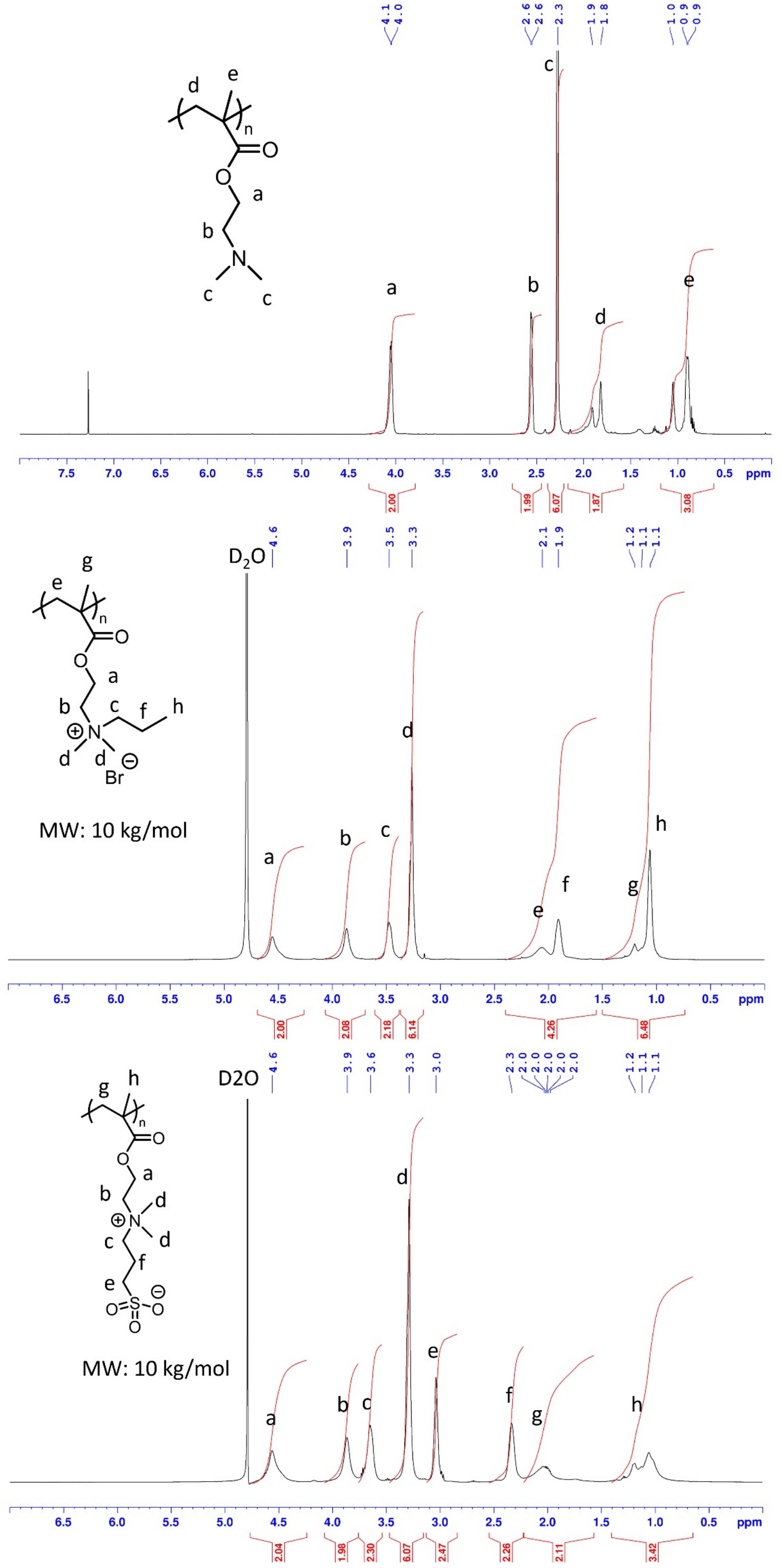

Figure S1: ^{1}H NMR results for PDMAEMA10k (top), PDMAEMA10k-electtrolyte (middle), and PDMAEMA10k-zwitterion (bottom).

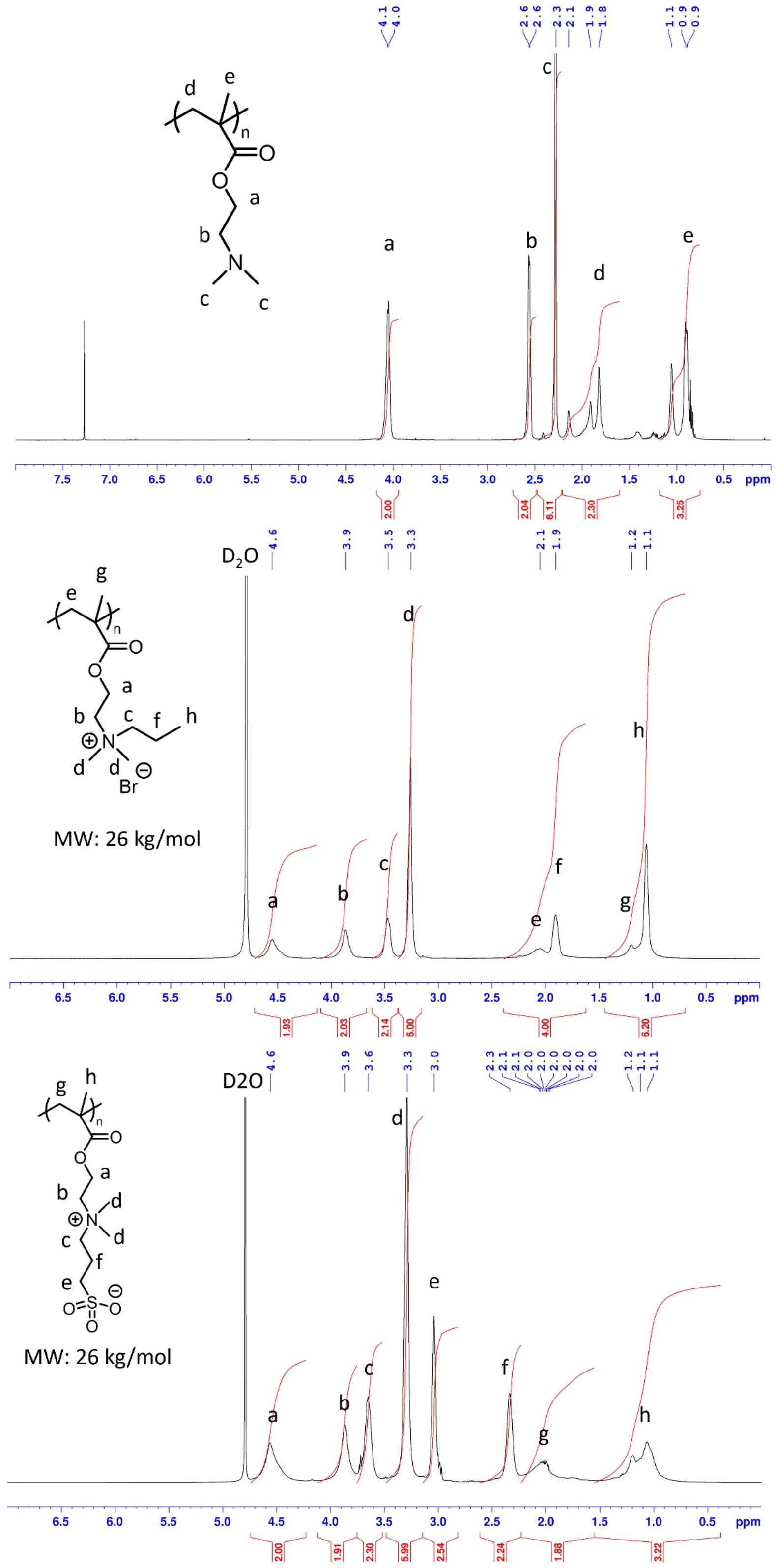

Figure S2: ^{1}H NMR results for PDMAEMA26k (top), PDMAEMA26k-electtrolyte (middle), and PDMAEMA26k-zwitterion (bottom).

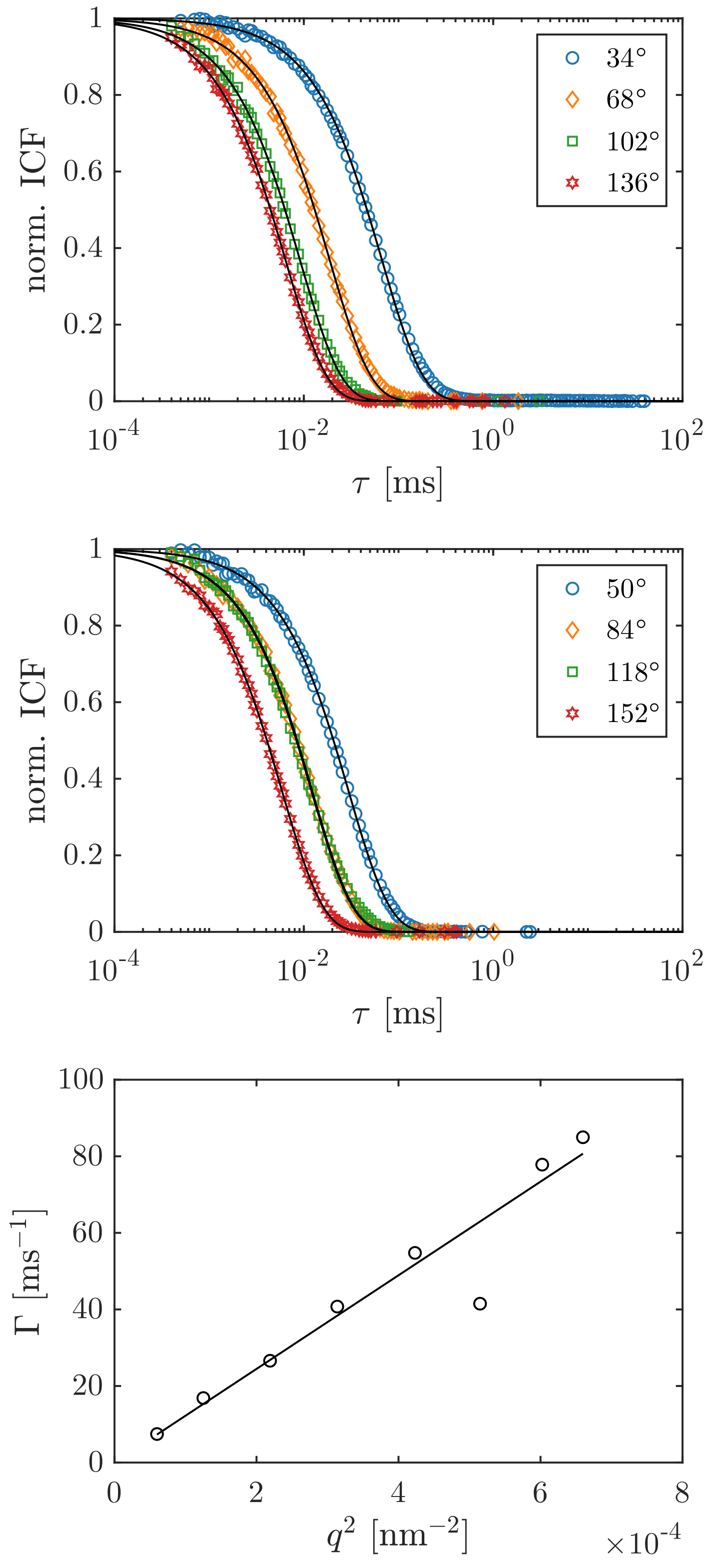

Figure S3: Top and middle: Multi-angle DLS results for the parent polymer, PDMAEMA10k are shown. Bottom: Decay rates plotted as a function of q^2 to determine diffusion constant.

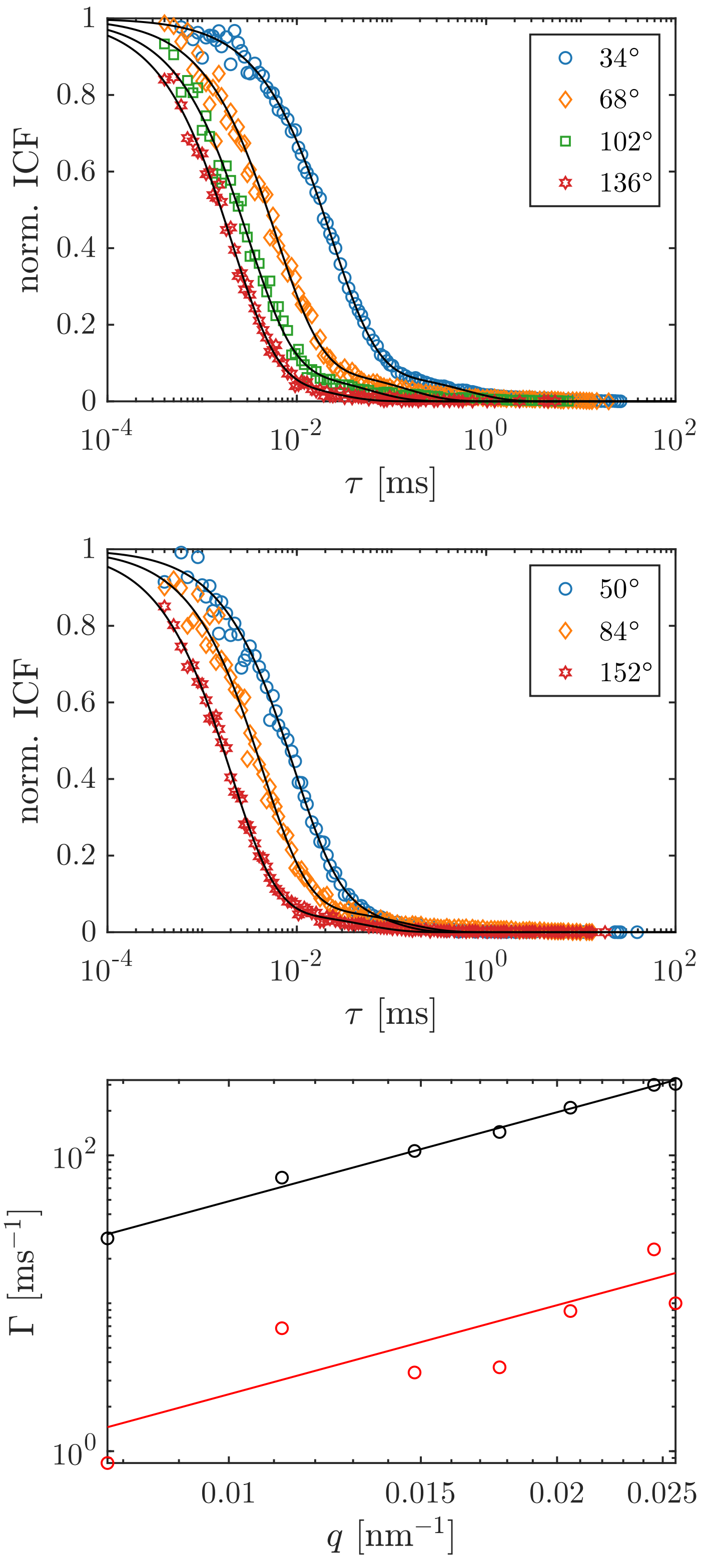

Figure S4: Top and middle: Multi-angle DLS results for PDMAEMA10k-electrolyte are shown. Bottom: Decay rates plotted as a function of q^2 to determine diffusion constant.

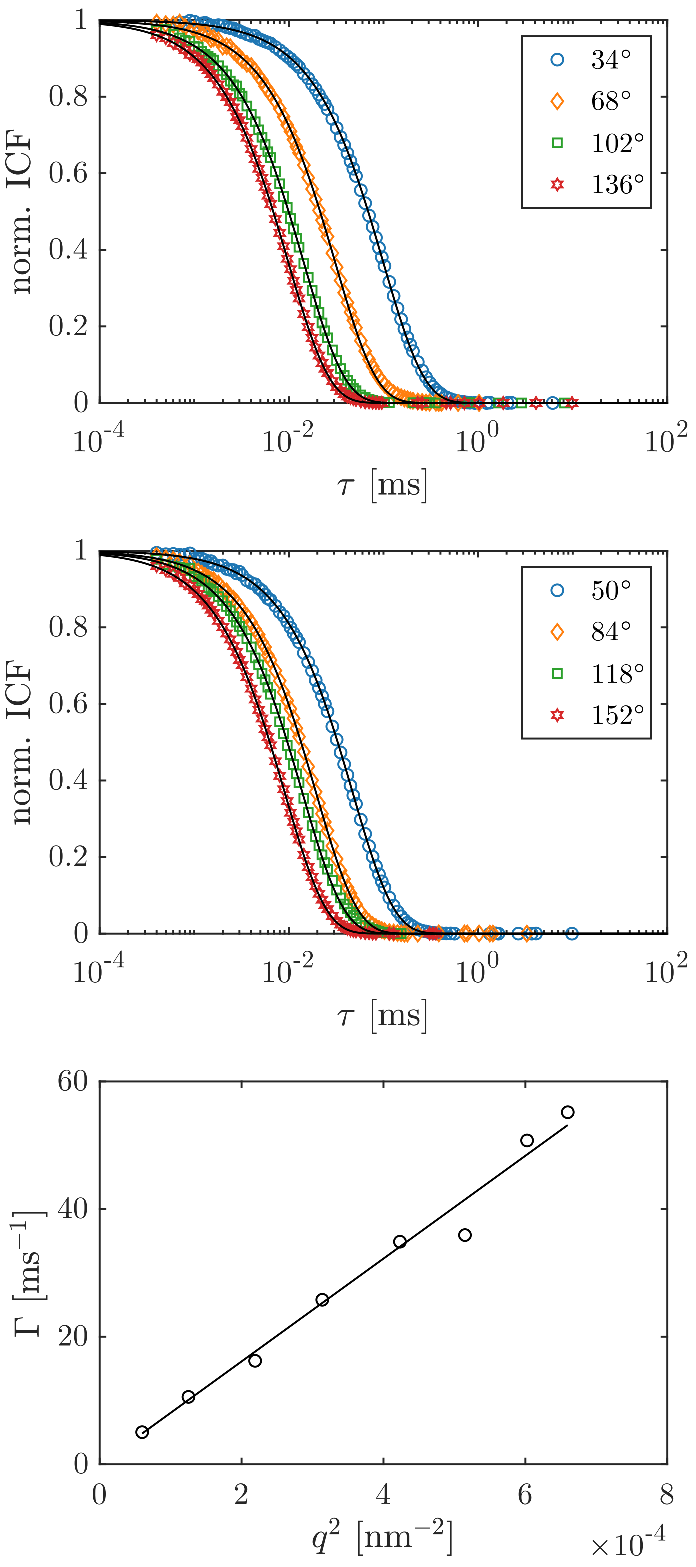

Figure S5: Top and middle: Multi-angle DLS results for the polyzwitterion, PDMAEMA10k-zwitterion are shown. Bottom: Decay rates plotted as a function of q^2 to determine diffusion constant.

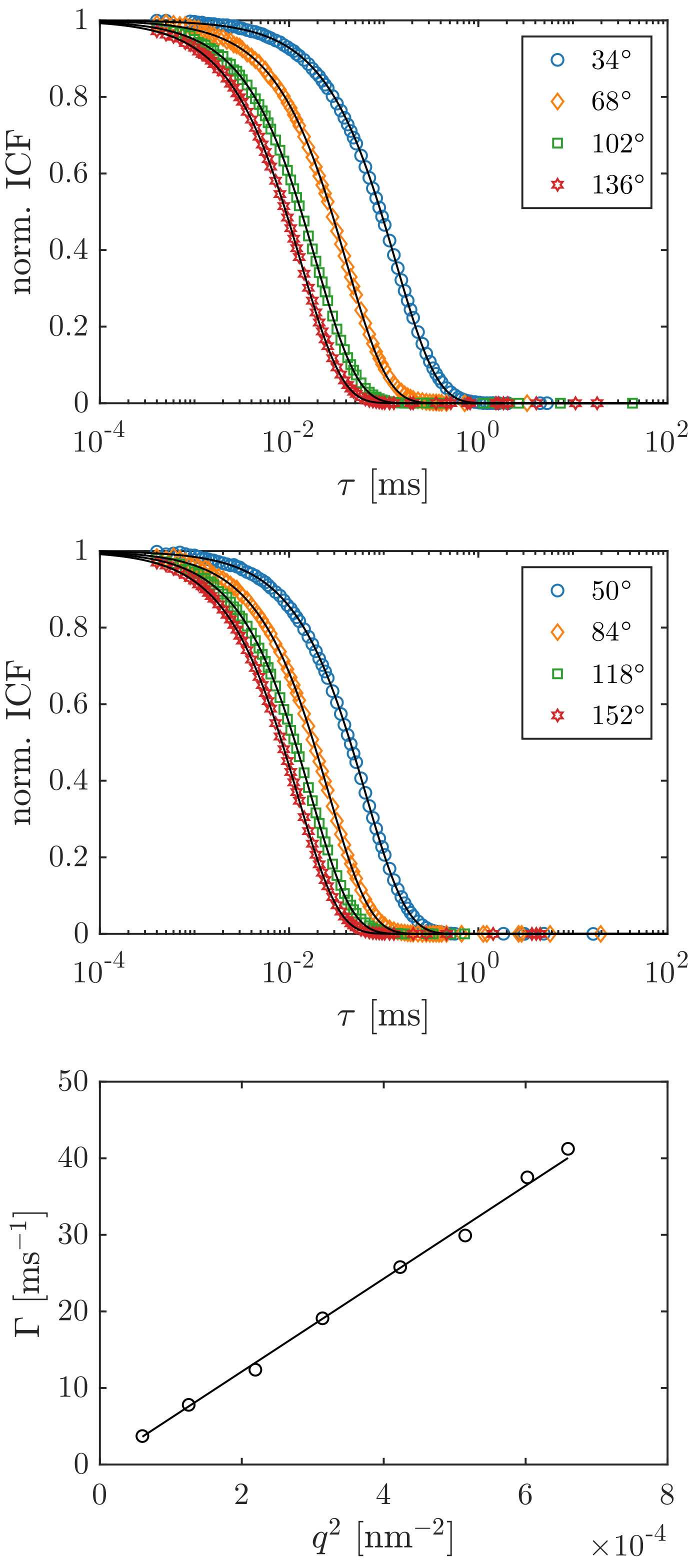

Figure S6: Top and middle: Multi-angle DLS results for the polyzwitterion, PDMAEMA10k-zwitterion with 0.01 M KBr are shown. Bottom: Decay rates plotted as a function of q^2 to determine diffusion constant.

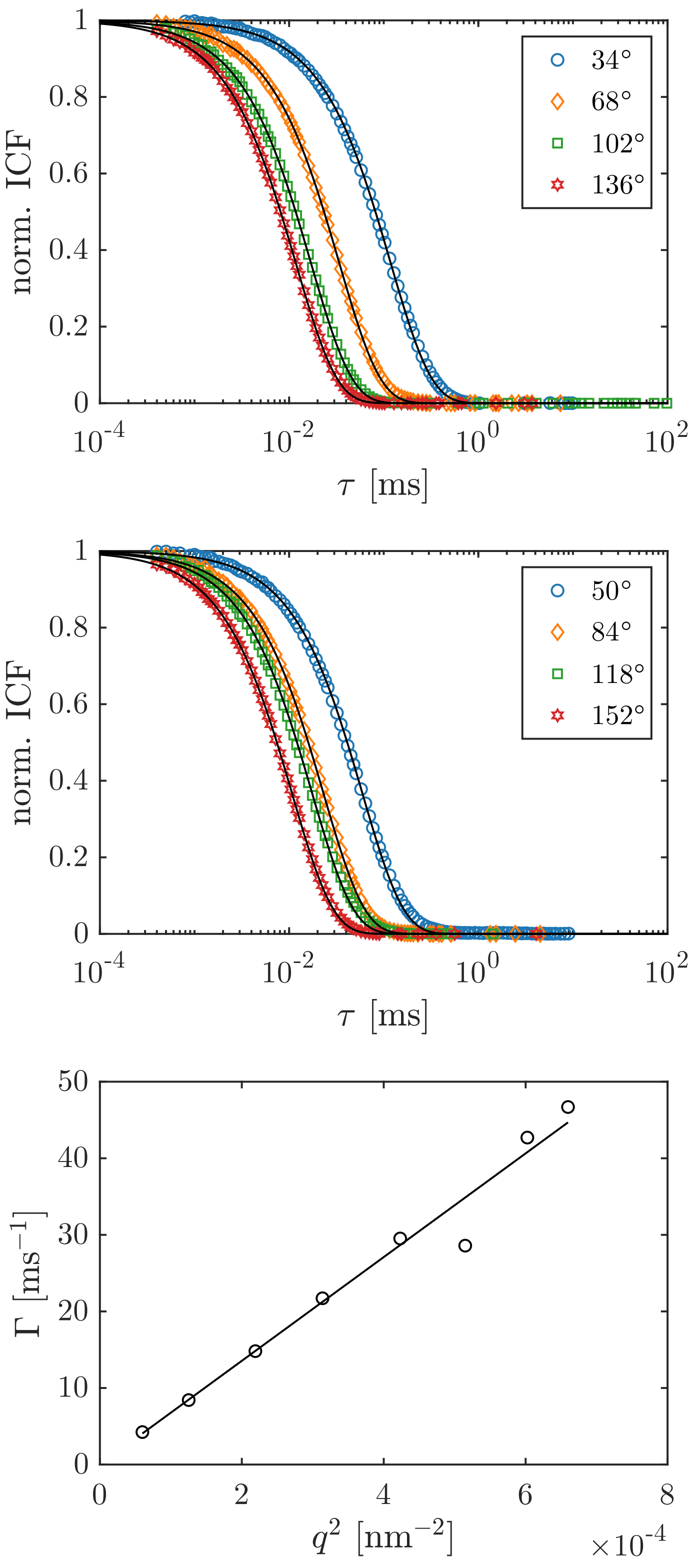

Figure S7: Top and middle: Multi-angle DLS results for the polyzwitterion, PDMAEMA10k-zwitterion with 0.1 M KBr are shown. Bottom: Decay rates plotted as a function of q^2 to determine diffusion constant.

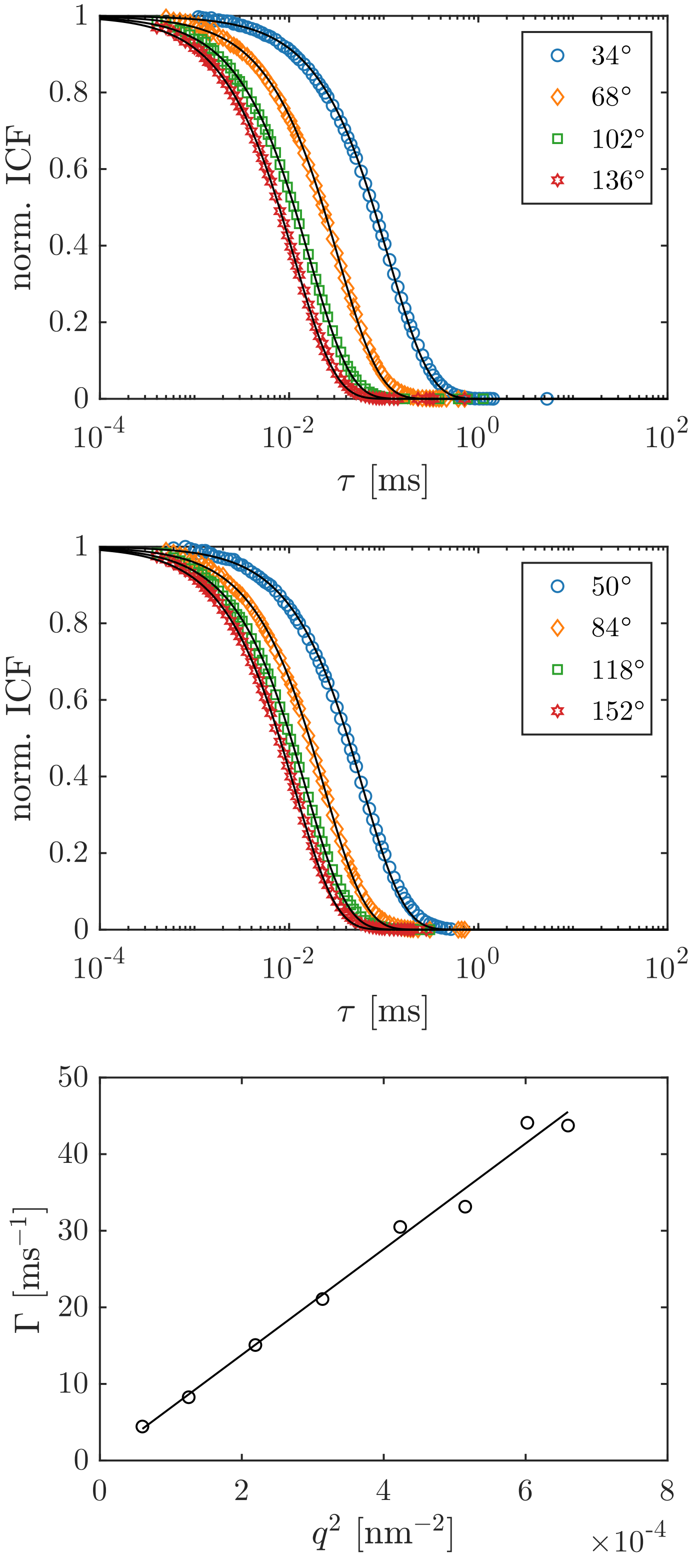

Figure S8: Top and middle: Multi-angle DLS results for the polyzwitterion, PDMAEMA10k-zwitterion with 0.5 M KBr are shown. Bottom: Decay rates plotted as a function of q^2 to determine diffusion constant.

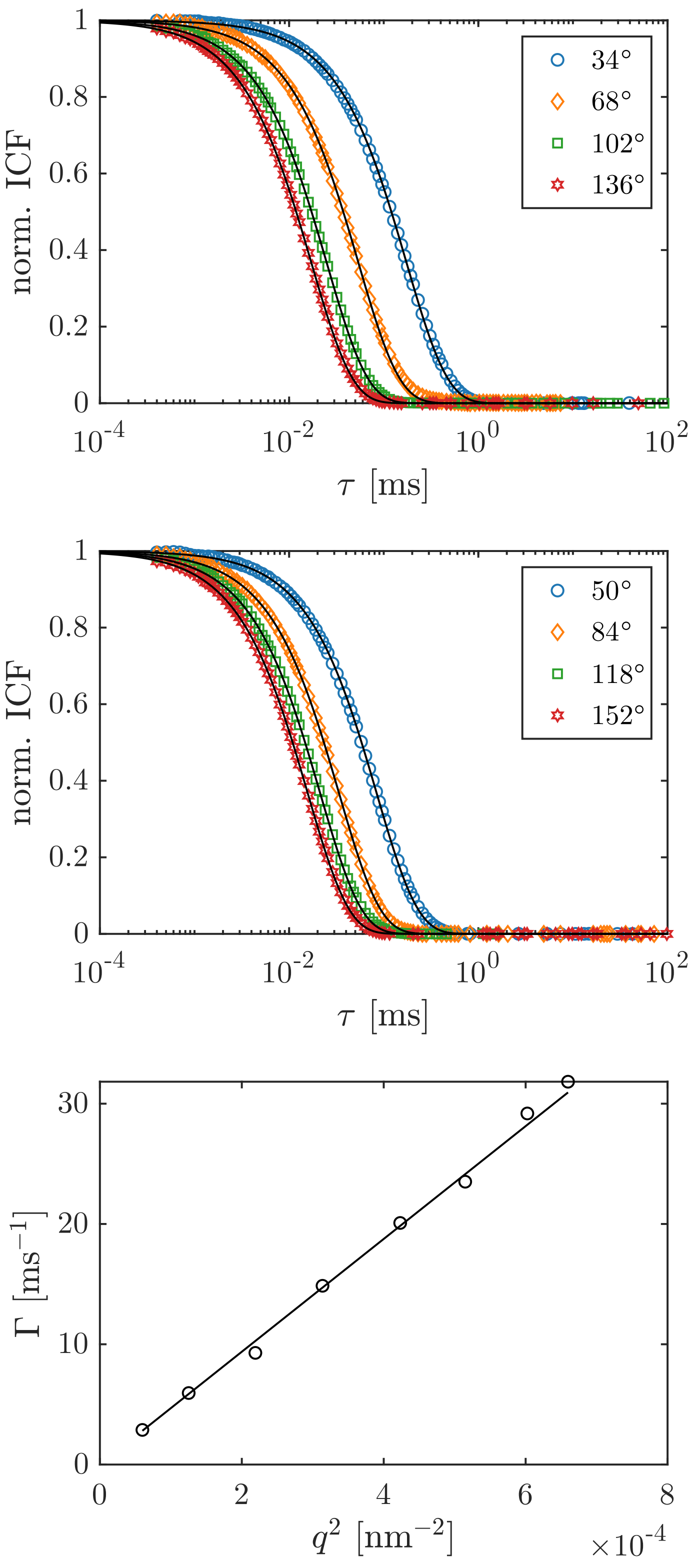

Figure S9: Top and middle: Multi-angle DLS results for the polyzwitterion, PDMAEMA26k-zwitterion with 0.01 M KBr are shown. Bottom: Decay rates plotted as a function of q^2 to determine diffusion constant.

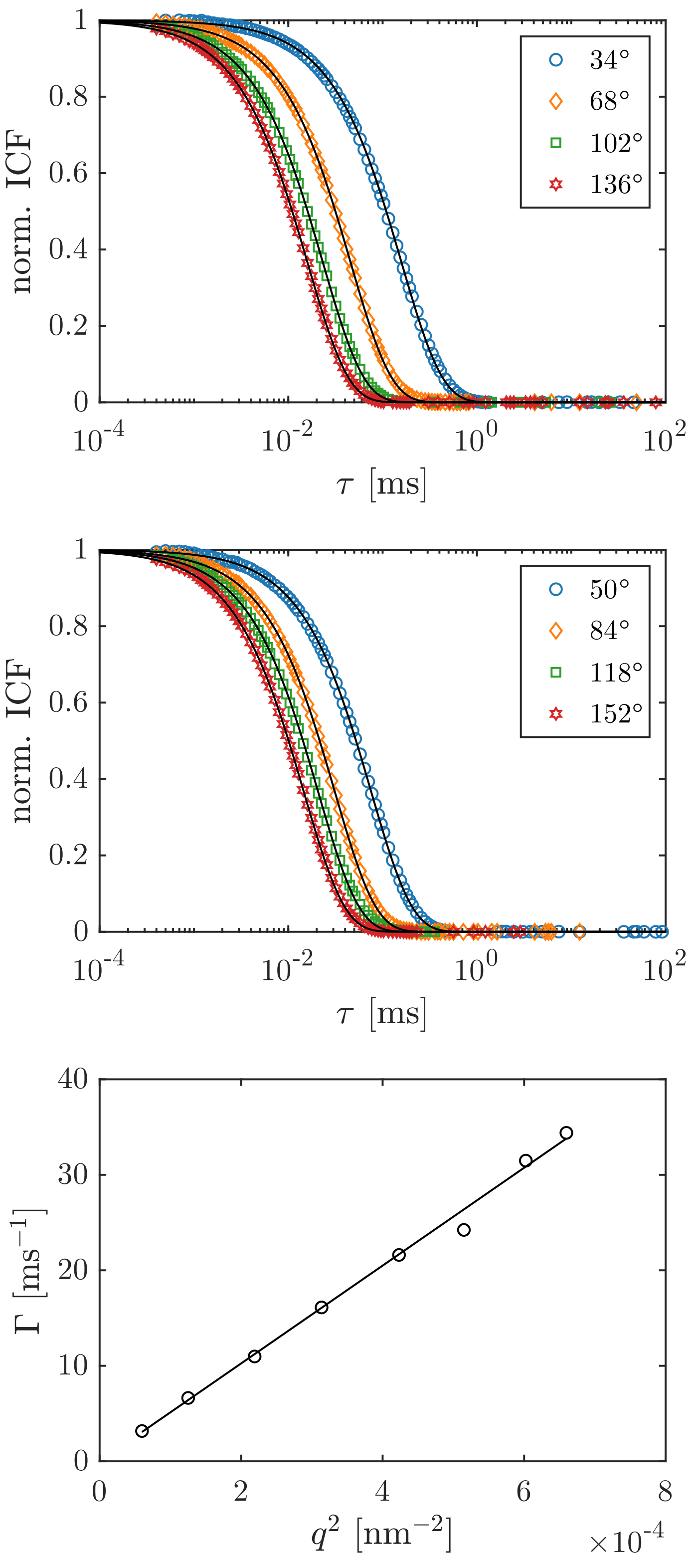

Figure S10: Top and middle: Multi-angle DLS results for the polyzwitterion, PDMAEMA26k-zwitterion with 0.1 M KBr are shown. Bottom: Decay rates plotted as a function of q^2 to determine diffusion constant.

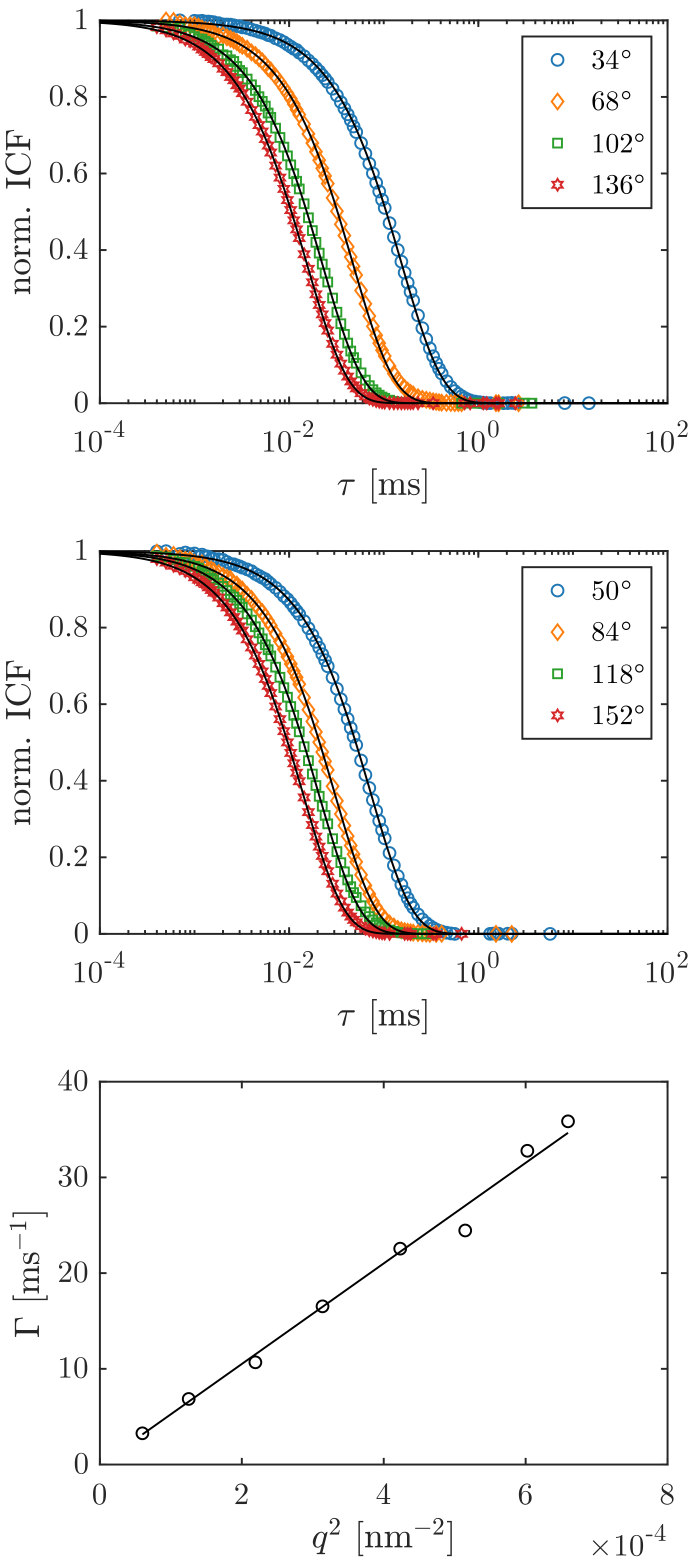

Figure S11: Top and middle: Multi-angle DLS results for the polyzwitterion, PDMAEMA26k-zwitterion with 0.5 M KBr are shown. Bottom: Decay rates plotted as a function of q^2 to determine diffusion constant.

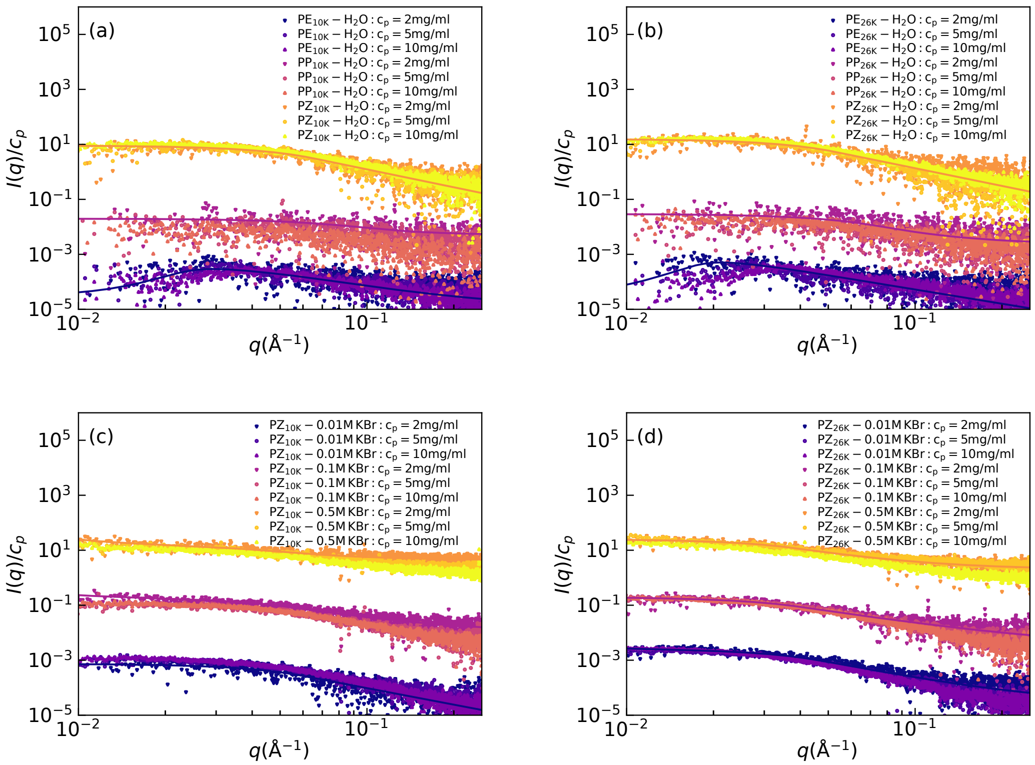

Figure S12: (a) SAXS results for the 10 K polymers studied in this work at three different concentrations of polymers in water. (b) SAXS results for the 26 K polymers studied in this work at three different concentrations of polymers (c_p) in water. (c) SAXS results for the 10K polymers studied in this work at three different concentrations of polymers in water containing KBr. (d) SAXS results for the 26 K polymers studied in this work at three different concentrations of polymers in water containing KBr.
